# High Glucose and Advanced Glycation End Products Induce CD147-Mediated MMP Activity in Human Adipocytes

**DOI:** 10.3390/cells10082098

**Published:** 2021-08-16

**Authors:** Abeer M. Mahmoud, Mohamed M. Ali

**Affiliations:** 1 Division of Endocrinology, Diabetes, and Metabolism, Department of Medicine, College of Medicine, University of Illinois at Chicago, Chicago, IL 60612, USA; 2 Department of Physical Therapy, College of Applied Health Sciences, University of Illinois at Chicago, Chicago, IL 60612, USA; mali37@uic.edu

**Keywords:** CD147, matrix metalloproteinases, adipocytes, diabetes, glycosylation, advanced glycation end-products

## Abstract

Basigin (CD147) is a transmembrane glycoprotein that regulates several physiological processes, including the production and activity of matrix metalloproteinases (MMPs). The activity of CD147 depends mainly on its glycosylation, which varies among pathophysiological conditions. However, it is unknown whether CD147 activity or its function in MMP regulation are affected by the diabetic environment, which is characterized by high glucose (HG) levels and an excess of glycation end products (AGEs). In this study, we investigated the effect of HG and AGEs on CD147 expression in human adipocytes. We also examined the mediating role of nuclear factor kappa B (NFκB) and receptor of AGE (RAGE) to this effect. Our findings show that carboxymethyl lysine and HG increased CD147 expression and glycosylation, which was accompanied by increases in MMP2 and MMP9 expression and activity, as well as upregulations of the *N*-acetylglucosaminyltransferase, MGAT5. These effects were abolished by NFκB and RAGE inhibition, *CD147* gene silencing, and by the glycosylation inhibitor, tunicamycin. In conclusion, the current findings indicate that AGEs and HG induce CD147 expression and glycosylation in adipocytes, with possible mediation by NFκB and RAGE. One of the critical outcomes of this pathway is augmented MMP activity known to contribute to cardiovascular complications in diabetes.

## 1. Introduction

Diabetes is a growing global epidemic and healthcare burden. The cardiovascular risk associated with diabetes is well-documented, yet contributing pathophysiological mechanisms are still to be elucidated [1]. Upregulated Matrix metalloproteinase (MMP) expression and activity contribute to the development of cardiovascular diseases (CVDs) such as atherosclerosis and hypertension [2]. MMPs are a group of enzymes that are responsible for extracellular matrix degradation and tissue remodeling. Increased expression of MMPs was detected in atherosclerotic plaques [3], and higher levels of circulating MMPs have been reported in patients with acute myocardial infarction, unstable angina, and hypertension [2]. Thus, MMPs and proteins that are responsible for their regulation are potential therapeutic targets that may reduce cardiovascular risk and require thorough investigations. On top of these regulators is CD147, which is also known as extracellular matrix metalloproteinase inducer (EMMPRIN) or basigin [4].

CD147 is a transmembrane glycoprotein that becomes active when highly glycosylated and participates in several physiological processes [5]. CD147 was initially reported in cancer and implicated in the enhanced ability of invasion and metastasis in some tumors [6]. It was also shown to be dysregulated in other pathological conditions such as atherosclerosis [7] and liver cirrhosis [8]. The upregulated MMP activity showed a positive correlation with increased CD147 expression in atherosclerotic plaques and circulating leukocytes of coronary artery disease patients [9,10]. The variability in CD147 glycosylation was found to modulate its function. The highly glycosylated form of CD147 has mainly been reported to induce MMP expression and activity [5]. However, the upstream regulation of CD147 expression, glycosylation, and interaction with the surrounding microenvironment in different tissues are not completely understood. Furthermore, whether CD147 glycosylation is enhanced in the diabetic milieu is uncertain.

A previous study demonstrated inductions of MMP expression and activity in monocytes in response to excess glucose [11]. The mechanism for this observation was thought to be mediated via CD147 upregulation. Yet, a similar mechanism in adipocytes has not been reported. In diabetes, chronic hyperglycemia results in the generation of AGEs that were found to be responsible for several complications associated with diabetes, such as tissue damage and vascular dysfunction [12]. AGEs exert their function either directly or via binding to their receptor, RAGE [13]. In either case, AGEs activate oxidative stress and NFκB-mediated inflammation [14]. We have previously reported higher levels of AGEs and RAGE in tissues from diabetic patients compared to lean healthy controls [15]. However, the link to CD147 expression and glycosylation and the downstream MMP induction are not thoroughly investigated. Therefore, in the current study, we examined the effect of AGEs and high glucose concentrations on the expression and glycosylation status of CD147 in human adipocytes. Furthermore, the mechanism by which this effect is achieved was examined.

## 2. Materials and Methods

### 2.1. Culture of Primary Subcutaneous Adipocytes

**Figure 1:**
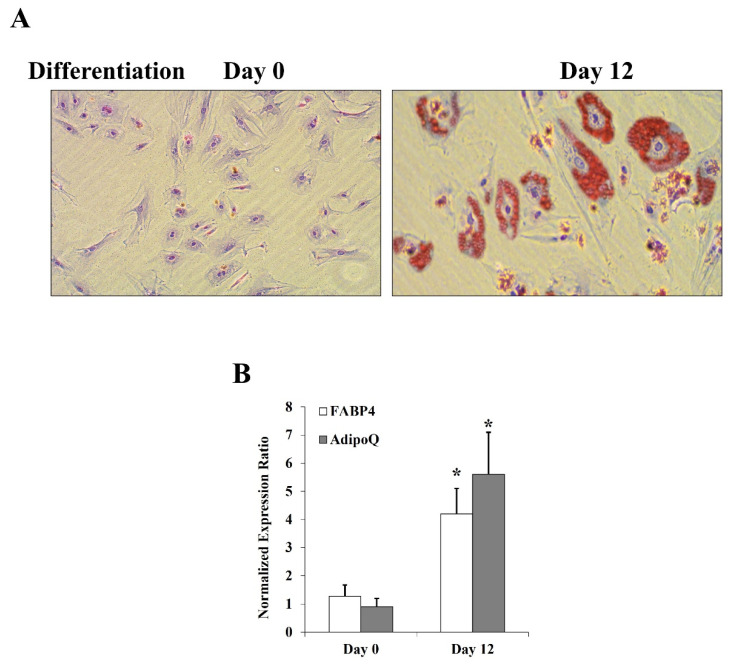
Assessment of adipocyte differentiation. (**A**) Microscopic images that demonstrate cytoplasmic accumulation of lipid droplets in adipocytes by differentiation days 0 and 12. (**B**) Quantitative measurements of *FABP4* and *AdipoQ* mRNA levels in differentiated adipocytes using real-time PCR. Results represent the means ± SD for three independent experiments, (* *p* < 0.05) for comparing day 12 with day 0.

Primary human preadipocytes were obtained from ScienCell Research Laboratories (Carlsbad, CA, USA). Cells were cultured for 48 h in Preadipocyte Medium (PAM) which contains Dulbecco’s Modified Eagle Medium (DMEM)/Ham’s F-12 (1:1, *v*/*v*), 4-(2-hydroxyethyl)-1-piperazineethanesulfonic acid (HEPES) pH 7.4, 5% fetal bovine serum (FBS), penicillin, streptomycin, and amphotericin B. Cells were maintained at 37 °C and 5% CO_2_ in a humidified incubator. For cell differentiation into mature adipocytes, cells were cultured for 12 days in Preadipocyte Differentiation Medium (PADM) that contains DMEM/Ham’s F-12 (1:1, *v*/*v*), HEPES pH 7.4, 5% FBS, biotin, pantothenate, human insulin, dexamethasone 3-isobutyl-1-methylxanthine (IBMX) peroxisome proliferator-activated receptor gamma (PPARγ) agonist, penicillin, streptomycin, and amphotericin B. Finally, cells were maintained in differentiation maintenance medium. This medium consists of DMEM/Ham’s F-12 (1:1, *v*/*v*), HEPES pH 7.4, 5% FBS, biotin, pantothenate, human insulin, dexamethasone, penicillin, streptomycin, and amphotericin B. All cell culture media were purchased from ScienCell Research Laboratories. To confirm differentiation, cells were stained with Oil Red O (BioVision Inc., Milpitas, CA, USA). Briefly, after washing with 1X PBS, cells were fixed in 10% formalin for 1 h, washed with distilled water, and incubated with 60% isopropanol for 5 min followed by Oil Red O Stain for 10 min. After stain removal, cells were washed with distilled water until the excess stain was completely removed. For counterstaining, cells were incubated in hematoxylin for 60 s, washed several times with distilled water, and imaged with an inverted microscope (Olympus, Tokyo, Japan). Differentiated cells (at day 12) showed cytoplasmic accumulations of fat globules compared to the undifferentiated state on day 0 (Figure 1A). Also, mRNA levels of *AdipoQ* and *FABP4* genes were measured via Real-time PCR (described below). Both genes were significantly upregulated in differentiated adipocytes (Figure 1B).

### 2.2. Adipocyte Treatments

Cells were cultured in Heracell CO_2_ incubator (Thermo Fisher Scientific, Waltham, MA, USA) at 37 °C, 20% O_2_, and 5% CO_2_. Cells were treated with different combinations of high glucose (25 mM), Carboxymethyl lysine-Bovine Serum Albumin (CML-BSA) (2 µg/mL), RAGE antibody (20 µg/mL), NFκB inhibitory peptide (15 µg/mL), and tunicamycin (15 μg/mL). Bovine serum albumin (BSA) and carboxymethyl lysine (CML) were purchased from Sigma-Aldrich (St. Louis, MO, USA). RAGE antibody and NFκB inhibitory peptide were acquired from R&D Systems (Minneapolis, MN, USA) and Novus Biologicals (Littleton, CO, USA), respectively. For CML-BSA preparation, 10 mg/mL of endotoxin-free BSA were prepared in PBS and incubated at 37 °C for six weeks with D-glucose (90 gm/L) in azide-containing PBS, as previously described in Chang et al. [16]. The degree of glycation was assessed by measuring fluorescence (excitation wavelength: 370 nm and emission wavelength: 440 nm) using a multimode plate reader (M3 Molecular Devices, San Jose, CA, USA). The CML content was also detected via a CML competitive ELISA assay (Cell Biolabs Inc., San Diego, CA, USA). Fluorescence intensity and CML content increased five-fold and ten-fold, respectively, in CML-BSA compared to BSA control. Cells incubated in BSA, DMSO (dimethyl sulfoximine), or mouse IgG (20 µg/mL) as negative controls for CML-BSA, NFκB inhibitory peptide and tunicamycin, and RAGE antibody, respectively. All experiments were run in triplicates and the results were presented as average ± standard deviation (SD).

### 2.3. CD147 and MGACT5 Gene Silencing

Differentiated adipocytes were cultured in 6-well plate at a concentration of 2 × 10^5^ cells per well for 24 h, after which cells were transfected with a pool of three *CD147*-specific small interfering RNAs (siRNAs) designed to induce effective knockdown of the *CD147* gene or the *MGACT5* gene (Santa Cruz Biotechnology, Dallas, TX, USA). Lipofectamine 2000 was used as the transfection reagent. Standard protocols for transient siRNA transfection with lipofectamine were followed. Briefly, lipofectamine was diluted in Opti-MEM medium (1:50). siRNAs (1 µg) were also diluted in Opti-MEM medium. Lipofectamine and siRNAs were mixed, incubated together for 20 min at room temperature, then the mixture was added to cells and incubated for 7 h The transfection media were then replaced with fresh growth media. The efficiency of gene downregulation was evaluated via measuring the mRNA levels of the corresponding genes using PCR assays.

### 2.4. Real-Time PCR

Total RNA was isolated from cultured cells using RNeasy kits from Qiagen (Germantown, MD, USA). The quantity and quality of RNA were assessed via NanoDrop OneC Microvolume UV-Vis Spectrophotometer (Thermo Fisher Scientific, Waltham, MA, USA). RNA was reverse transcribed into cDNA using iScript cDNA Synthesis Kit from Bio-Rad Laboratories (Hercules, CA, USA) followed by determination of mRNA expression of target genes via real-time RT-PCR (CFX96 RT PCR Detection System, Bio-Rad Laboratories, Hercules, CA, USA). In these experiments, SsoAdvanced™ Universal SYBR^®^ Green Supermix, SYBR Green (Bio-Rad Laboratories, Hercules, CA, USA) was used. Gene-specific primers were designed using primer3 software v. 0.4.0 (http://bioinfo.ut.ee/primer3-0.4.0/ accessed on 1 May 2021) and manufactured by Invitrogen Life Technologies (Carlsbad, CA, USA). Table 1 shows DNA sequences for the designed primers. *GAPDH* was used as the housekeeping gene, and the normalized expression ratio of each target was calculated using the 2-∆∆Ct (Livak method) [17]. All reactions were performed in triplicates from three independent experiments, and results are presented as averages ± SD.

### 2.5. Western Blotting

Total protein was extracted using RIPA lysis buffer that consists of 20 mM Tris-HCl (pH 7.5), 150 mM NaCl, 1 mM Na2 EDTA, 1 mM EGTA, 1% NP-40, 1% sodium deoxycholate, 2.5 mM sodium pyrophosphate, 1 mM b-glycerophosphate, 1 mM Na3VO4, and 1 µg/mL leupeptin (Cell Signaling, Danvers, MA, USA) supplemented with protease and phosphatase inhibitor cocktail (MS-SAFE) from Sigma-Aldrich. Total protein concentration was quantified via Pierce BCA Protein Assays (Thermo Fisher Scientific, Waltham, MA, USA). Ten μg of total protein were electrophoresed by 4–12% Bis-Tris gradient gels (Bio-Rad Laboratories, Hercules, CA, USA) and transferred to Polyvinylidene fluoride (PVDF). Membranes were subsequently incubated in primary antibodies for CD147 (rabbit monoclonal from Abcam), MMP2, and MMP9 (mouse monoclonal from Santa Cruz Biotechnology, Dallas, TX, USA) overnight at 4 °C followed by incubation with Horseradish Peroxidase (HRP)-conjugated secondary antibodies (Santa Cruz Biotechnology, Dallas, TX, USA) for 1 h at room temperature. GAPDH (Cell Signaling, Danvers, MA, USA) was used as a loading control. ECL Western blotting detection reagents (Amersham Biosciences, Piscataway, NJ, USA) followed by x-ray film development were used for signal identification. The X-ray films were scanned and quantified via NIH Image J software.

### 2.6. ELISA Assays

Soluble CD147, MMP2, and MM9 were measured in cell culture media using a specific Quantikine ELISA Kit (R&D Systems) for each protein. The manufacturer’s protocols were followed. Briefly, media were passed through 0.2 μm depth filters to get rid of cells and particles. Samples, standards, and controls were added to the 96-well plate and incubated for 2 h at room temperature on an orbital shaker. This was followed by four steps of washing and incubation with 200 μL of the specific protein conjugate for 2 h at room temperature on a shaker. Washing steps were repeated, followed by incubation with 200 μL of Substrate Solution for 30 min at room temperature protected from light. Finally, the reaction was terminated by adding 50 μL of Stop Solution, and the absorbance was measured at a wavelength of 450 nm with the additional measurement at 570 nm for plate imperfection correction.

### 2.7. Immunoprecipitation and Glycoprotein Staining

CD147 protein was extracted from cell culture media using Dynabeads protein G magnetic beads and following the recommended protocol (Invitrogen, Life Technologies). In brief, a specific monoclonal antibody for CD147 (ab108308) from Abcam was incubated with Dynabeads for 60 min at room temperature followed by incubation with 2 mL of the collected cell culture media at 4 °C overnight. Proteins were eluted from the complex using SDS-PAGE sample buffer followed by gel electrophoresis and glycoprotein staining using Pierce™ Glycoprotein Staining Kit (Thermo Fisher Scientific). Gels were immersed in 50% methanol (100 mL) for 30 min, followed by 3% acetic acid (100 mL) for 10 min. Gels were washed with distilled water and incubated with Oxidizing Solution (25 mL) for 15 min. Gels were then transferred to Glycoprotein Stain (25 mL) for 15 min with gentle agitation followed by incubation with Reducing Solution (25 mL) for 5 min. Finally, gels were washed thoroughly with 3% acetic acid and then with distilled water and glycoproteins appeared as magenta bands. Gels were scanned and quantified for CD147 glycosylation (band intensities) via the NIH ImageJ software.

### 2.8. MMP2/9 Activity Assay

InnoZyme™ Gelatinase (MMP-2/MMP-9) Fluorogenic Activity Assay kit (Millipore Sigma, Burlington, MA, USA) was used to assess the activity of MMPs2 and MMP9 in cell culture media. This assay utilizes a triple-helical collagen-like peptide that is highly selective for MMP2 and MMP9. This approach relies on the fluorescence resonance energy transfer (FRET) phenomenon. The fluorescence of the FRET peptide is quenched while it is intact and released only when the peptide is cleaved. Accordingly, this peptide is used as an indicator of MMP2/9 activity. The manufacturer protocol was followed. Briefly, conditioned media from different treatments were passed through 0.2 μm depth filters to get rid of cells and debris and then adjusted to the same protein concentration in all samples. Pro-MMP2 and pro-MMP9 were pre-activated by incubation with p-Aminophenylmercuric Acetate (APMA) followed by incubation with the substrate at 37 °C for 6 h. Finally, fluorescence was measured via multimode plate reader (M3 Molecular Devices) with excitation and an emission wavelength of 320 and 405 nm, respectively. 

### 2.9. Statistical Analysis

All results were representative of triplicates from three independent experiments and expressed as average ± SD. Data were analyzed using Student’s t-test or one-way ANOVA followed by a post hoc analysis (Bonferroni’s) when appropriate. Results were considered statistically significant when the *p*-value was less than 0.05.

## 3. Results

### 3.1. Effects of AGEs and HG on CD147 Expression and Glycosylation in Adipocytes

**Figure 2:**
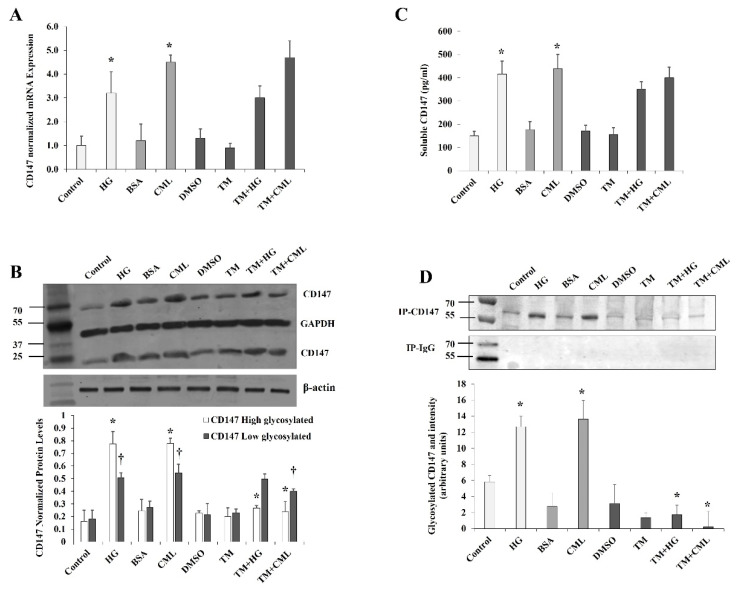
Effects of HG and CML on CD147 expression and glycosylation in differentiated adipocytes. Cells were treated with HG (25 mM) or CML (2 µg/mL) with and without tunicamycin (TM, 15 μg/mL) for 24 h. Normal glucose-containing media (control), BSA, and DMSO were used as controls for HG, CML, and TM treatments, respectively. (**A**) Quantitative assessment of *CD147* mRNA levels normalized to the house keeping gene, *GAPDH* using real-time PCR. (**B**) Western blot analysis and quantification of the normalized signal intensity of CD147 proteins; GAPDH and β-actin were used as housekeeping proteins. (**C**) Measurements of soluble CD147 protein in cell culture media using CD147 Quantikine ELISA assay. (**D**) Representative image and quantification of CD147 protein glycosylation using Pierce™ Glycoprotein Stain. Mouse IgG was used as a control for the immunoprecipitation step. The graphs represent the means ± SD for 3 independent experiments. (* *p* < 0.05) for comparison with the corresponding control (HG vs. control; CML vs. BSA; TM + HG vs. HG; and TM + CML vs. CML). († *p* < 0.05) for comparison with the corresponding control in low glycosylated protein (**B**).

Differentiated adipocytes were cultured for 24 h in high glucose (HG, 25 mM) or CML-BSA (2 µg/mL) for which normal glucose (5 mM) and BSA were used as controls, respectively. Our data showed upregulations of *CD147* mRNA (HG: ↑ by 3.2 folds, CML: ↑ by 4.5 folds, *p* < 0.001, Figure 2A). Similarly, protein levels of CD147 increased in response to HG by 4.8 folds for the high glycosylated form (M.W. ~60 kDa) and 2.8 folds for the low glycosylated form (M.W. ~27 kDa) compared to controls. In response to CML, CD147 increased by 3.2 folds for the high glycosylated form and 2.0 folds for the low glycosylated form (*p* < 0.001, Figure 2B). These results indicate an increase in the glycosylated form of CD147 to a greater extent than the low glycosylated form in response to both HG and CML. This pattern was reversed by simultaneous incubation with the glycosylation inhibitor, tunicamycin (Figure 2B). At the used concentration, tunicamycin had no effect on global protein synthesis as measured by the GAPDH and β-actin housekeeping proteins.

The transmembrane form of CD147 could be cleaved to produce a soluble form that has been described as a biomarker in certain cancers and chronic diseases [18,19]. Thus, we sought to measure the soluble form of CD147 in the adipocyte culturing media in response to 24 h incubation with HG and CML. To this end, quantitative CD147-specific ELISA assays were used. We observed a significant rise in soluble CD147 protein in the preconditioned media that were collected from HG- and CML-treated cells compared to control adipocytes (HG: ↑ by 2.8 folds, CML: ↑ by 2.5 folds, *p* < 0.001, Figure 2C).

Next, we investigated the effect of HG and CML on CD147 glycosylation via immunoprecipitation of CD147 from total cell lysate followed by gel electrophoresis and in-gel staining of glycosylated protein with periodic acid-Schiff (PAS) stain. Using this method, we observed an increase in CD147 glycosylation in response to HG (2.2 folds *p* < 0.001) and CML (4.9 folds, *p* < 0.000) treatments (Figure 2D). This effect was inhibited by tunicamycin.

**Figure 3:**
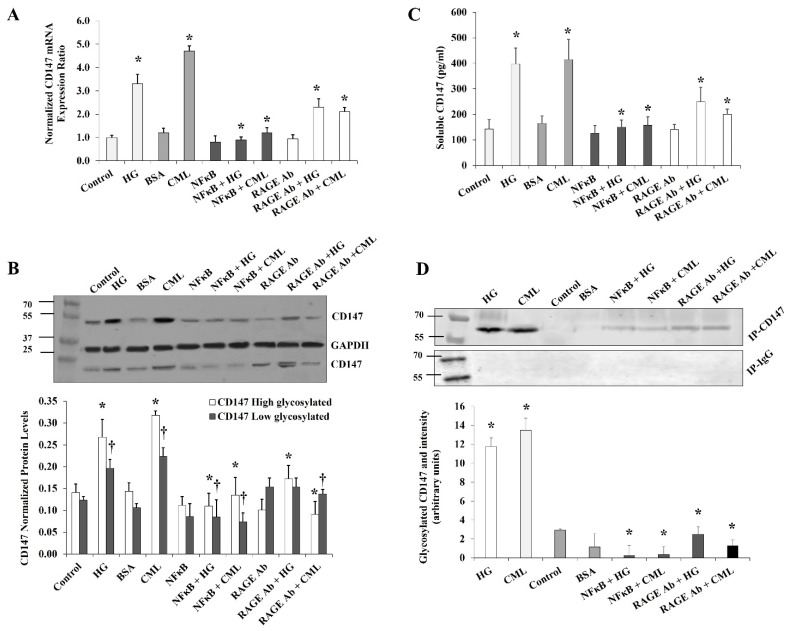
Effects of inhibiting NFκB and RAGE on HG- and CML-mediated changes in differentiated adipocytes. Cells were treated with HG (25 mM) or CML (2 µg/mL) with and without NFκB inhibitory peptide (15 µg/mL) or RAGE antibody (20 µg/mL) for 24 h. (**A**) Quantitative assessment of *CD147* mRNA levels normalized to the house keeping gene, *GAPDH* using real-time PCR. (**B**) Western blot analysis and quantification of the normalized signal intensity of CD147 proteins. (**C**) Measurements of soluble CD147 protein in cell culture media using CD147 Quantikine ELISA assay. (**D**) Representative image and quantification of CD147 protein glycosylation using Pierce™ Glycoprotein Stain. Mouse IgG was used as a control for the immunoprecipitation step. The graphs represent the means ± SD for three independent experiments. (* *p* < 0.05) for comparison with the corresponding control (HG vs. control; CML vs. BSA; NFκB + HG vs. HG; and NFκB + CML vs. CML; RAGE Ab + HG vs. HG; and RAGE Ab+CML vs. CML). († *p* < 0.05) for comparison with the corresponding control in low glycosylated protein (**B**).

It was previously shown that the effects of hyperglycemia and AGEs are mediated, at least in part, by the transcription factor, NFκB. It has also been proposed that AGEs may activate NFκB either directly or mediated by the RAGE receptor [12]. In order to investigate the contribution of NFκB and RAGE in the observed effect of HG and CML on CD147 expression, cells were preincubated with either the NFκB inhibitory peptide (15 µg/mL) or RAGE antibody (20 µg/mL) for 2 h before the incubation with HG and CML. Cells incubated with DMSO or mouse IgG were used as negative controls. NFκB inhibition reduced HG- and CML-mediated induction of *CD147* mRNA and protein levels by 59–72% and 56–74%, respectively (Figure 3A,B). On the other hand, RAGE blocking decreased CML-induced effects on *CD147* mRNA and protein expression by 36–72% with a relatively lower impact on HG-induced outcomes (25–35%, Figure 3A,B). We also observed that NFκB and RAGE inhibition significantly reduced the HG- and CML-induced release of soluble CD147 protein in the media (Figure 3C). In addition, NFκB and RAGE inhibition interfered with HG and CML-induced CD147 glycosylation (Figure 3D). These data might indicate a significant contribution of NFκB and RAGE to the increased expression and glycosylation of CD147 under conditions of hyperglycemia and excess AGEs.

**Figure 4:**
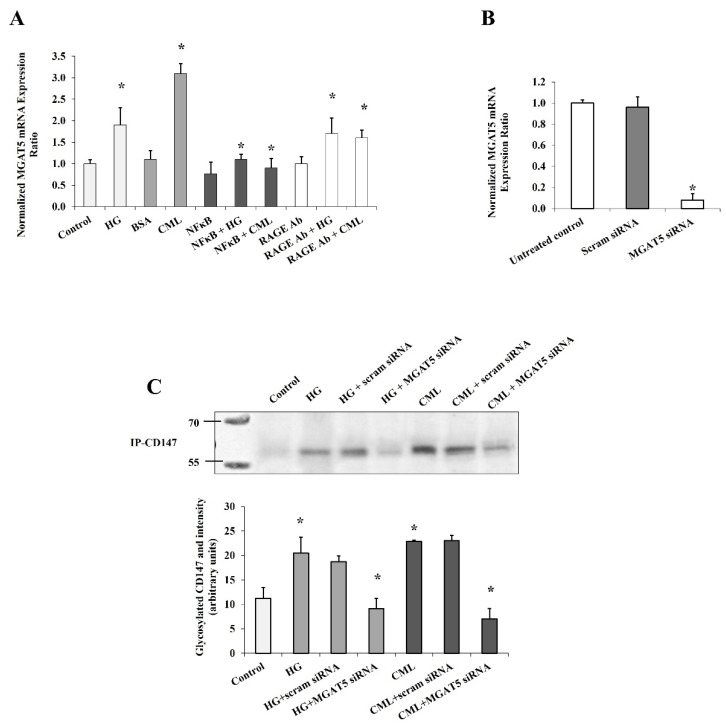
The mediating role of *MGAT5* to HG and CML effects on CD147 glycosylation. Quantitative assessment of *MGAT5* mRNA levels in cells treated with HG (25 mM) or CML (2 µg/mL) with and without NFκB inhibitory peptide (15 µg/mL), RAGE antibody (20 µg/mL) (**A**), or *MGAT5*-specific siRNAs (**B**), using real-time PCR. (**C**) Representative image and quantification of CD147 protein glycosylation in cells treated with HG (25 mM) or CML (2 µg/mL) with and without *MGAT5*-specific siRNAs using Pierce™ Glycoprotein Stain. The graphs represent the means ± SD for 3 independent experiments. (* *p* < 0.05) for comparison with the corresponding control.

In order to elucidate this mechanism a step further, we measured the mRNA expression of *MGAT5* (alpha-1,6-mannosylglycoprotein 6-beta-*N*-acetylglucosaminyltransferase) that has been reported to play a major role in CD147 glycosylation [20]. *MGAT5* gene expression increased significantly in response to HG and CML treatment (90% and 2.8 folds, respectively, Figure 4A). This effect was markedly lost after NFκB inhibition and RAGE blocking, suggesting that the *MGAT5* gene expression is a downstream target to the NFκB/RAGE pathway. To test if *MGAT5* was a mediator for the effect of HG and CML on CD147 glycosylation in adipocytes, we used a set of siRNAs that was found to be effective in downregulating *MGAT5* mRNA expression (Figure 4B). Indeed, the increased CD147 glycosylation observed in adipocytes in response to HG and CML treatment was significantly nullified when *MGAT5* was downregulated (Figure 4C).

### 3.2. Effects of AGEs and HG on MMP Expression and Activity in Differentiated Adipocytes

Matrix metalloproteinases (MMPs) are a group of enzymes that are responsible for degradation of the extracellular matrix and connective tissue proteins and play a critical role in vascular remodeling [21]. A growing body of evidence supports the involvement of dysregulated MMPs in cardiovascular disease (CVD) including atherosclerosis, aneurysms, and hypertension [2]. The effect of CD147, also known as EMMPRIN (Extracellular matrix metalloproteinase inducer) on inducing MMP expression and activity is well-established especially in the context of cancer progression [22]. However, this pathway is not well characterized in adipose tissues, and whether adipocytes are a source of active MMPs in response to hyperglycemia and glycation products has yet to be determined.

**Figure 5:**
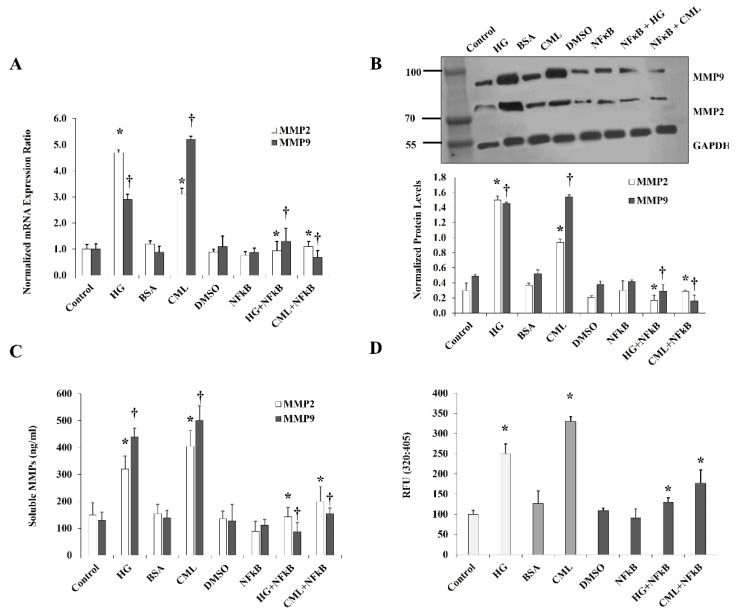
Effects of HG and CML on MMP2 and MMP9 expression and activity in differentiated adipocytes. Cells were treated with HG (25 mM) or CML (2 µg/mL) with and without NFκB inhibitory peptide (15 µg/mL) for 24 h. (**A**) Quantitative assessment of *MMP2* and *MMP9* mRNA levels normalized to the house keeping gene, *GAPDH* using real-time PCR. (**B**) Western blot analysis and quantification of the normalized signal intensity of MMP2 and MMP9 proteins. (**C**) Measurements of soluble MMP2 and MMP9 proteins in cell culture media using corresponding Quantikine ELISA assays. (**D**) Quantitative assessment of MMP2/9 activity using InnoZyme™ Gelatinase (MMP2/MMP9) Fluorogenic Activity Assay. Results represent the means ± SD for three independent experiments. (* *p* < 0.05) for MMP2 comparison with the corresponding control (HG vs. control; CML vs. BSA; NFκB+HG vs. HG; and NFκB + CML vs. CML). († *p* < 0.05) for MMP9 comparison with the corresponding control.

For the effect of HG and CML on MMPs, we measured the expression and activity of MMP2 and MMP9 since these MMPs have been reported to be abundantly expressed in adipocytes [23]. Cells were treated with HG or CML for 24 h, followed by analyses of MMP2 and MMP9 protein and mRNA levels. Preconditioned cell culture media were also collected and examined for MMP2 and MMP9 activity via Gelatinase (MMP-2/MMP-9) Activity Assays. Our results show that HG and CML induced the mRNA levels of *MMP2* (4.7 folds and 2.6 folds) and *MMP9* (2.9 folds and 5.9 folds), respectively, compared to controls (Figure 5A). Similar to their effect on mRNA, HG and CML increased protein levels of MMP2 (5.0 folds and 2.6 folds) and MMP9 (2.9 folds and 3 folds), respectively (Figure 5B). Media concentrations of MMP2 and MMP9 were also shown to rise in response to HG and CML compared to untreated cells (Figure 5C). Cells treated with HG or CML showed increases in MMP2 and MM9 activity by 2.5 folds and 2.6 folds, respectively (Figure 5D). All effects of HG and CML on MMPs were abolished by inhibiting NFκB via a specific inhibitory peptide (Figure 5A–D).

**Figure 6:**
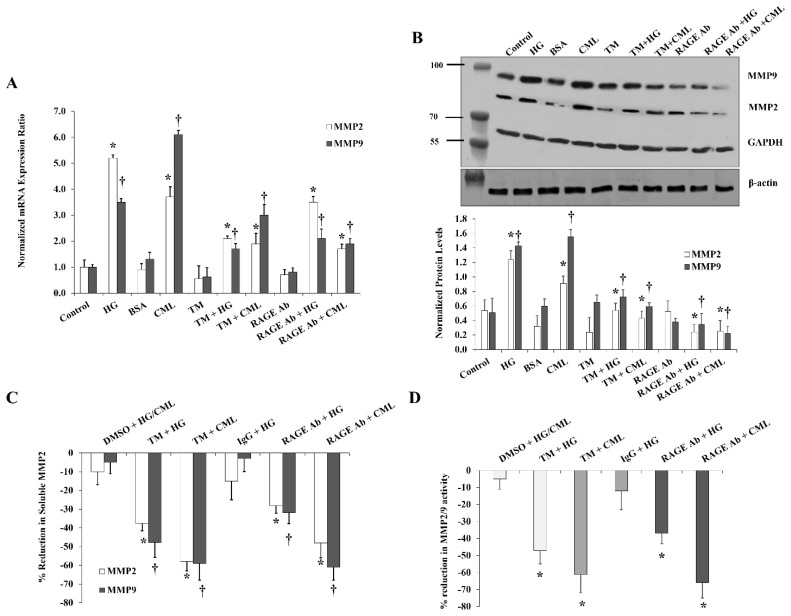
Effects of inhibiting glycosylation and blocking RAGE on HG- and CML- mediated changes in MMP2 and MMP9. Cells were treated with HG (25 mM) or CML (2 µg/mL) with and without tunicamycin (TM, 15 μg/mL) or RAGE antibody (20 µg/mL) for 24 h. (**A**) Quantitative assessment of *MMP2* and *MMP9* mRNA levels normalized to the house keeping gene, *GAPDH* using real-time PCR. (**B**) Western blot analysis and quantification of the normalized signal intensity of MMP2 and MMP9 proteins. GAPDH and β-actin were used as housekeeping proteins (**C**) Percentage reduction in soluble MMP2 and MMP9 proteins relative to the corresponding control (HG or CML). (**D**) Percentage reduction in MMP2/9 activity relative to the corresponding control (HG or CML). Results represent the means ± SD for three independent experiments. (* *p* < 0.05) for MMP2 (MMP activity in (**D**)) comparison with the corresponding control (HG vs. control; CML vs. BSA; TM + HG vs. HG; and TM + CML vs. CML; RAGE Ab + HG vs. HG; and RAGE Ab + CML vs. CML). († *p* < 0.05) for MMP9 comparison with the corresponding control.

Blocking RAGE with a specific antibody reduced the effect of HG and CML on MMP2/9 expression and activity in a similar way to inhibiting NFκB. RAGE blocking reduced the mRNA and protein levels by 33% and 55% for MMP2 and by 40% and 33% for MMP9 compared with the HG treatment (Figure 6A,B). The soluble fraction of MMP2 and MMP9 decreased by 28% and 32%, respectively relative to the HG condition (Figure 6C). Furthermore, the HG-induced MMP2/9 activity was reduced by 37% (Figure 6D). Similar changes were observed for the CML effects on MMPs after RAGE blocking (Figure 6A–D). Tunicamycin, which was proven in our experiments to diminish CD147 glycosylation, also suppressed the increases in MMP2/9 expression and activity in response to HG and CML (Figure 6A–D) confirming the role of the glycosylated CD147 in MMP induction.

**Figure 7:**
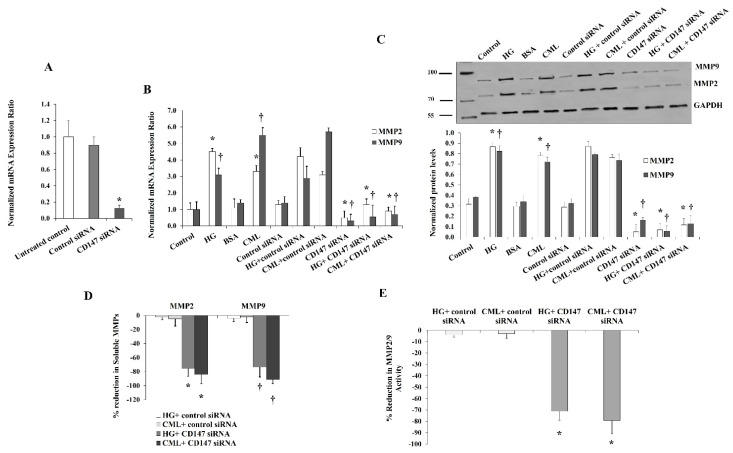
Effects of *CD147* silencing on HG- and CML- mediated changes in MMP2 and MMP9. Cells were treated with HG (25 mM) or CML (2 µg/mL) with and without *CD147* siRNA for 24 h. *Control (non-specific)* siRNA was used as a negative control for *CD147* siRNA. (**A**,**B**) Quantitative assessment of *MMP2* and *MMP9* mRNA levels normalized to the house keeping gene, *GAPDH* using real-time PCR. (**C**) Western blot analysis and quantification of the normalized signal intensity of MMP2 and MMP9 proteins. (**D**) Percentage reduction in soluble MMP2 and MMP9 proteins relative to the corresponding control (HG or CML). (**E**) Percentage reduction in MMP2/9 activity relative to the corresponding control (HG or CML). Results represent the means ± SD for three independent experiments. (* *p* < 0.05) for MMP2 (MMP activity in (**E**)) comparison with the corresponding control (HG vs. control; CML vs. BSA; *CD147* siRNA + HG vs. HG; and *CD147* siRNA + CML vs. CML). († *p* < 0.05) for MMP9 comparison with the corresponding control.

To further verify the contribution of CD147 in mediating the effect of HG and CML on MMP expression and activity, we utilized a specific set of *CD147*-targeting siRNAs that was validated as an effective tool for downregulating CD147 mRNA (Figure 7A). Our results showed that the increases in *MMP2* and *MMP9* mRNA and protein levels in response to HG and CML were reduced to a great extent under conditions of *CD147* silencing compared to the siRNA controls (Figure 7A,B). We also observed significant reductions (up to 80%) in the soluble fractions of MMP2/9 measured in cell culture media (Figure 7C). Marked reductions were also observed in the MMP2/9 activity measured via the InnoZyme™ Gelatinase Assay (Figure 7D) in cells transfected with *CD147* siRNAs.

## 4. Discussion

Diabetes is characterized by increased biochemical stress and injury at the cellular level due to an excess of circulating glucose and biochemical compounds such as AGEs [12,24]. Chronic hyperglycemia and exposure to AGEs modify protein glycosylation profiles, and the best-known example for this is the glycosylated hemoglobin (HbA1C). The degree of glycosylation determines the activity of some proteins, such as the MMP inducer, CD147. The latter is altered in pathologic conditions such as neurodegenerative diseases and cancer [25]. However, this pathway has not been fully investigated in adipocytes residing in the diabetic milieu. Accordingly, in the current study, we investigated the effect of CML and HG on CD147 status in human differentiated adipocytes. The major finding in the present study is that CD147 expression and glycosylation were induced in adipocytes cultured under conditions of HG and CML compared to the control conditions. This outcome was accompanied by increased expression and activity of MMP2/9 and suggested to be mediated by NFκB/RAGE pathway.

The MMP inducer, CD147 is an *N*-glycosylated protein that exists in two forms, a low glycosylated form that has a molecular weight of ~30 kDa and a high glycosylated form that has a molecular weight between 40 and 60 kDa. Previous studies that used different *N*-glycan inhibitors suggested that the *N*-glycosylation of CD147 constitutes almost half of the molecule’s size and contributes significantly to its function. Aberrant expression and glycosylation of CD147 have been reported to carry significant implications in cancers such as hepatoma and leukemia and determine the stability of atherosclerotic plaques [5,26]. These observations highlight the clinical significance of this protein as a therapeutic target.

In protein *N*-glycosylation, carbohydrates (also referred to as glycans) bind to the nitrogen of an asparagine residue of a protein. This process depends on substrate availability and activity of enzymes such as glycosyltransferases and glycosidases. Protein *N*-glycosylation is a dynamic process that reflects health status and adaptations to the external environment and extracellular milieu, which makes it a perfect target for future preventive and therapeutic interventions. The process of *N*-glycosylation starts with the conversion of glucose to glucose-6-P by the enzyme hexokinase. A fraction of the glucose-6-P is converted to glucose-1-P by the enzyme phosphoglucomutase, and this sugar form interacts with UTP forming the high-energy donor UDP-Glc. The latter is used to synthesize dolichol-P-glucose, which is the first step in *N*-linked glycan biosynthesis [27]. Accordingly, it is conceivable that a high glucose level could contribute to aberrant glycosylation. In support of this assumption, a study by Hoffmann et al. [28] reported that hyperglycemia-induced glycosylation in vascular endothelial cells of murine models contributes significantly to the progression of diabetic complications. Moreover, previous studies such as the EPIC-Potsdam Cohort [29] and the Finland Cardiovascular Risk Study (FINRISK) [30] have demonstrated that diabetic patients have higher levels of protein glycosylation and that the degree of protein glycosylation is proportional to glucose concentrations and predictive of diabetes-related vascular complications.

Due to its inherent glycosylation properties, CD147 is a candidate for modifications caused by HG or excess AGEs that accompany diabetes and metabolic diseases. Thus, studying CD147 expression and glycosylation provides valuable insight into the development of diabetic complications and how to target them therapeutically. Bao et al. [11] reported inductions of CD147 glycosylation in monocytes treated with high glucose or AGEs, which they suggested to be mediated by a pathway that involves tumor necrosis factor-alpha (TNFα) and transforming growth factor bets (TGFβ). In the current study, we observed similar changes in CD147 expression and glycosylation in adipocytes. The contributing mechanism in our study was found to be mediated by the NFκB/RAGE pathway. Previous studies have shown, in various in vitro and murine models, that HG and AGEs are capable of inducing inflammation and oxidative stress via activating the NFκB transcription factor [31,32,33,34,35]. It has been shown that AGEs are capable of activating NFκB via RAGE-dependent and independent pathways [31,36,37]. It was also demonstrated that RAGE and NFκB are involved in a positive feedback cycle where NFκB activates the transcription of RAGE, and the latter, in turn, triggers the activation of NFκB via the ERK/MAPK (extracellular signal-regulated kinases/mitogen-activated protein kinases) pathway [38,39,40]. In the current study, both NFκB inhibition and RAGE blocking suppressed the achieved induction in CD147 expression and glycosylation in response to HG and CML. These findings suggest a mediating role of NFκB to the HG and CML-induced effects on CD147 expression. In support of this, previous studies demonstrated a similar regulatory role of NFκB in CD147 expression [11,41]. Yet, the mechanism by which NFκB modifies CD147 glycosylation is not equally understood. Herein, we proposed a role of the glycosyltransferase, MGAT5 (*N*-acetylglucosaminyltransferase V) that has been previously shown to be under NFκB transcriptional regulation [42]. We observed upregulations in *MGAT5* mRNA in response to HG and CML, which was lost after inhibiting the NFκB/RAGE pathway. These observations are consistent with previous studies that reported the role of NFκB in glycan synthesis via regulating the transcription of several glycosyltransferases and glycosidases either directly or via downstream transcription factors such as ETS1 [42].

The role of glycosylation in CD147-mediated induction of MMPs has been inconsistent in the literature. Some studies have demonstrated that glycosylation is a required posttranslational modification for CD147 to exert its effect on MMP expression and activity. In these studies, the function of CD147 in inducing MMP expression and activity was lost in response to glycosylation inhibitors such as tunicamycin [22,43]. Other studies reported that the non-glycosylated recombinant CD147 protein was capable of inducing MMP expression [19]. Nevertheless, these studies were conducted extracellularly, and they do not entirely reflect intracellular biological processes. It is also worth noting that different cell types have varying degrees of CD147 glycosylation, and the extent to which CD147 activity is dependent on glycosylation in different tissues and cells remains to be further investigated [5]. In our study, we observed a loss of CD147-dependent MMP expression and activity in adipocytes in response to the glycosylation inhibitor, tunicamycin. Also, *CD147* silencing diminished the CD147-dependent elevation in MMP expression and activity, which confirms that this effect was mediated, at least in part, by CD147. Additionally, our studies showed that HG and CML increased the level of soluble CD147, an effect that could be secondary to the upregulated expression of CD147 proteins or a consequence of the enhanced protein-cleaving activity of MMPs. This subject was not further investigated in the current research. However, future investigations are required to elucidate this phenomenon, since it could serve as a valid target to reduce circulating CD147 that might exert its function in a paracrine manner, resulting in remote tissue remodeling and systemic inflammation.

Observations from previous clinical studies that reported upregulations of the CD147 in patients with acute myocardial infarction and ischemia/reperfusion injury lend clinical significance to this research [44,45,46]. This clinical significance is also supported by findings from epidemiological studies that indicated an association between circulating MMPs and the risk of developing cardiovascular diseases and clinical studies that reported higher levels of circulating MMPs in patients with atherosclerosis, coronary artery diseases, and diabetic cardiomyopathy [2,47]. Moreover, experimental studies showed that proatherogenic stimuli, such as oxidized LDL, increased CD147 expression in monocytes and subsequently increased monocyte migration and induced inflammation [48]. The current study is the first to demonstrate that adipocytes which grow in diabetic-like milieu exhibit inductions in CD147 expression and glycosylation accompanied by enhanced MMP expression and activity. This outcome is expected to cause tissue and vascular remodeling and augmented inflammation locally or systemically in case widespread tissues such as the adipose tissues are involved. Targeting this pathway may provide a valid mechanism for the transcriptional regulation of MMPs and point to CD147 as a potential therapeutic target in diabetes.

## Figures and Tables

**Table 1:** Sequences of Real-time PCR primers.

Gene Name	Orientation	Primer Sequence	Tm	Product Size
*CD147*	Fw-primer	TTCAGCCTCTGGGTCTGAGT	60.0	238
	Rv-primer	GCCAAGAGGTCAGAGTCGTC	60.0	
*MMP2*	Fw-primer	ACAGCAGGTCTCAGCCTCAT	60.0	151
	Rv-primer	TGAAGCCAAGCGGTCTAAGT	60.0	
*MMP9*	Fw-primer	TTGACAGCGACAAGAAGTGG	60.0	179
	Rv-primer	GCCATTCACGTCGTCCTTAT	60.0	
*FABP4*	Fw-primer	TACTGGGCCAGGAATTTGAC	60.0	181
	Rv-primer	GTGGAAGTGACGCCTTTCAT	60.0	
*AdipoQ*	Fw-primer	CCTAAGGGAGACATCGGTGA	60.0	173
	Rv-primer	GTAAAGCGAATGGGCATGTT	60.0	
*MGAT5*	Fw-primer	CCTTCCCCTTCTTTTCCAAG	60.0	235
	Rv-primer	CCTGGGAGCCAGTACACATT	60.0	
*GAPDH*	Fw-primer	CGACCACTTTGTCAAGCTCA	60.0	228
	Rv-primer	AGGGGTCTACATGGCAACTG	60.0	

## Data Availability

The data presented in this study are available in the article.
